# The Landscape of Monogenic Parkinson’s Disease in Populations of Non-European Ancestry: A Narrative Review

**DOI:** 10.3390/genes14112097

**Published:** 2023-11-17

**Authors:** Christos Koros, Anastasia Bougea, Athina Maria Simitsi, Nikolaos Papagiannakis, Efthalia Angelopoulou, Ioanna Pachi, Roubina Antonelou, Maria Bozi, Maria Stamelou, Leonidas Stefanis

**Affiliations:** 1 1st Department of Neurology, Eginition Hospital, National and Kapodistrian University of Athens, 11528 Athens, Greece; chkoros@gmail.com (C.K.); simitsh@yahoo.gr (A.M.S.); nickpap88@gmail.com (N.P.); angelthal@med.uoa.gr (E.A.); pachiioanna@gmail.com (I.P.); rantonelou@gmail.com (R.A.); lstefanis@bioacademy.gr (L.S.); 2 Dafni Psychiatric Hospital, 12462 Athens, Greece; mbozigr@yahoo.gr; 3 2nd Department of Neurology, Attikon Hospital, National and Kapodistrian University of Athens, 12462 Athens, Greece; 4 HYGEIA Hospital, 15123 Athens, Greece; mariastamelou@gmail.com

**Keywords:** Parkinson’s disease, genetic, non-European, Leucine Rich Repeat Kinase 2 gene, Glucocerebrosidase gene, Alpha-Synuclein gene, Parkin gene

## Abstract

Introduction: There has been a bias in the existing literature on Parkinson’s disease (PD) genetics as most studies involved patients of European ancestry, mostly in Europe and North America. Our target was to review published research data on the genetic profile of PD patients of non-European or mixed ancestry. Methods: We reviewed articles published during the 2000–2023 period, focusing on the genetic status of PD patients of non-European origin (Indian, East and Central Asian, Latin American, sub-Saharan African and Pacific islands). Results: There were substantial differences regarding monogenic PD forms between patients of European and non-European ancestry. The G2019S Leucine Rich Repeat Kinase 2 (LRRK2) mutation was rather scarce in non-European populations. In contrast, East Asian patients carried different mutations like p.I2020T, which is common in Japan. Parkin (PRKN) variants had a global distribution, being common in early-onset PD in Indians, in East Asians, and in early-onset Mexicans. Furthermore, they were occasionally present in Black African PD patients. PTEN-induced kinase 1 (PINK1) and PD protein 7 (DJ-1) variants were described in Indian, East Asian and Pacific Islands populations. Glucocerebrosidase gene variants (GBA1), which represent an important predisposing factor for PD, were found in East and Southeast Asian and Indian populations. Different GBA1 variants have been reported in Black African populations and Latin Americans. Conclusions: Existing data reveal a pronounced heterogeneity in the genetic background of PD. A number of common variants in populations of European ancestry appeared to be absent or scarce in patients of diverse ethnic backgrounds. Large-scale studies that include genetic screening in African, Asian or Latin American populations are underway. The outcomes of such efforts will facilitate further clinical studies and will possibly contribute to the identification of either new pathogenic mutations in already described genes or novel PD-related genes.

## 1. Introduction

Parkinson’s disease (PD) is usually sporadic, meaning that there is no family history. In approximately 10% of cases, however, there are affected relatives, which may implicate a hereditary-genetic form of the disease. Especially in patients of a young age (disease onset before 40 years of age), an underlying genetic cause is highly possible. Genetic forms may occur even in sporadic cases, again more commonly with a relatively early age of onset. In recent years, further genetic causes of PD have been discovered [1].

Such mutations are not particularly rare in certain populations. The clinical picture and course might vary depending on the exact genetic cause and the specific mutation, but there is also an intrafamilial variability. The genetic diagnosis of the disease is important in these cases not only for family planning and assessment of patient prognosis but also because it can be taken into account for specific therapeutic options, namely device-aided treatments [like Deep Brain Stimulation (DBS) or Levodopa-Carbidopa enteric gel pumps] and even more so for inclusion in emerging clinical studies that are applied to specific genetic forms of the disease, in the context of pharmacogenomics [2]. In the era of individualized therapies, the strategy of treatment in genetic PD forms may differ substantially compared to idiopathic PD [3].

The discovery of Parkinson’s disease (PD) related genes has revolutionized PD research during the last few years. Following the discovery of the SNCA gene in 1997 [4], an ever-increasing number of causative genes for PD have been described. Pathogenic mutations in autosomal-dominant genes like the α-synuclein (SNCA), leucine-rich repeat kinase 2 (LRRK2), and gene encoding for the vacuolar protein-sorting 35 ortholog (VPS35) and autosomal recessive genes like PRKN, PINK1 and DJ1 have been increasingly recognized as important genetic factors in young-onset PD or in cases of inheritance within families. The importance of the risk factor Glucocerebrosidase gene (GBA1), accounting for as many as 5–10% of PD cases, is outstanding. However, there has been a bias in existing literature data as most previous studies have assessed patient groups of European ancestry, mainly in Europe and North America. The majority of other races are underrepresented, including even different racial minorities of the aforementioned countries [especially in the United States or in European countries [5].

Our target was to review previously published research on the genetic background (variants of PD-causing genes) of PD populations of non-European or mixed ancestry. 

## 2. Materials and Methods

We reviewed articles published during the 2000–2023 period, focusing on genetic data in PD patients of non-European ancestry (East and Central Asian, Indian, Latin American, sub-Saharan African, and Pacific islands). We searched PUBMED, SCOPUS and EMBASE from 2000–2023 using the following terms: “Parkinson’s disease”, “genetic”, “non-Caucasian”, “non-European”, “East Asia”, “South East Asia” “Central Asia”, “India”, ”Sub-Saharan Africa”, “Latin America”, and “Pacific islands”. Our literature search focused on monogenic forms of PD, and as a result, common polymorphisms that increase PD risk or polygenic forms have generally not been included. Only articles published in English have been included in our present review.

## 3. Results

### 3.1. Autosomal-Dominant Genes

#### 3.1.1. LRRK2

Missense mutations in the LRRK2 (leucine-rich repeat kinase 2) gene segregated with the disease in cases with autosomal-dominant inheritance. Such mutations were first described by two different groups of PD patients [6]. LRRK2 mutations are thought to relate to a gain-of-function mechanism. LRRK2 is considered to regulate the phosphorylation of Rab GTPases, thus regulating vesicular trafficking [1,7]. The p.G2019S mutation is notably the most common mutation (up to 4% of familial cases and 1% of sporadic cases in a large worldwide study) [8]. The phenotype of affected patients resembles idiopathic PD. Additional pathogenic mutations include p.R1441G/C/H [6], p.Y1699C [6] and p.I2020T [9].

In a multicenter study, patients harboring a LRRK2 mutation exhibited a rather favorable motor course and preservation of cognition, as assessed through the use of the MiniMental State Examination (MMSE), compared to idiopathic PD [8,10]. Moreover, a number of LRRK2 carriers might exhibit hyposmia [8]. Interestingly, REM Sleep Behavior Disorder (RBD) appears to be rather rare [11].

**Figure 1:**
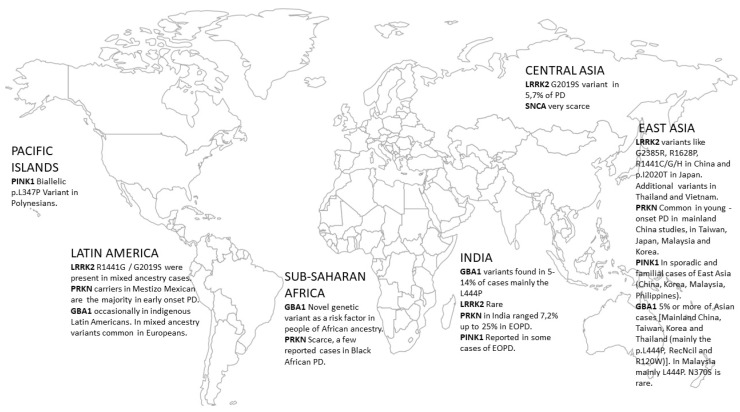
Map showing the worldwide distribution of PD-related gene variants in populations of non-European ancestry.

Regarding the epidemiological profile of LRRK2, the frequency of mutations is variable and related to both the geographical region and ethnicity assessed (Figure 1, Table 1).

A notable number of cases with familial and/or sporadic forms of the disease with the p.G2019S mutation has been reported in Ashkenazi Jews (up to 15%) and North African Arab-Berbers (up to 30–40%) [12,13]. In contrast, this variant is relatively rare in familial PD in different populations, such as in Greece [14].

From a global perspective, the G2019S LRRK2 mutation [prevalent in patients of European ancestry (notably the Iberian Peninsula) and Ashkenazi Jewish/Northern African ancestry] is probably due to a common founder effect that seems to be scarce in other populations, except for those of Central Asia. The G2019S LRRK2 mutation was identified in Uzbek patients with idiopathic and familial PD (5.7% and 17.6%, respectively) [15]. A Whole-Exome Sequencing (WES) study including 50 index individuals with young-onset PD from Kazakhstan yielded a low prevalence of PD-related genes with two non-related familial PD patients with the LRRK2 p.(Arg1441Cys) mutation, while a few additional cases had extremely rare variants of uncertain significance in LRRK2 and other genes [16].

Notably, East Asian patients carried different variants, mainly R1441G, R1441C, G2385R and p.I2020T in Japanese populations. G2385R and R1628P were reported mainly in East Asian countries, where they could be detected in ~5–10% of cases. These have been considered to act as genetic risk factors, almost doubling PD risk [17]. When evaluating East Asian populations, A419V, R1628P, and G2385R LRRK2 variants were prominent [18]. In a large Japanese study, 1402 PD patients were assessed (sporadic PD *n* = 653 and familial PD *n* = 749). In total, 23 patients harbored pathogenic LRRK2 variants (four with p.R1441G, five with p.R1441H, seven with p.G2019S, and seven with p.I2020T), while additional patients carried other rare variants. Additional risk variants, p.P1446L and p.G2385R, were also detected in two groups of 10 and 146 patients. [17]. In a second study from Japan, LRRK2 was a prevalent genetic factor in all age groups because of the importance of the Asian-specific variants such as LRRK2 p.G2385R [19].

In a Vietnamese cohort, the most common genetic variant was LRRK2 c.4883G>C (p.Arg1628Pro), and patients with PD harboring this variant were found to have a distinct phenotype [20]. LRRK2 p.R1628P (c.4883G>C) is associated with PD in Chinese and Thais. In a study from Thailand, 485 PD patients and 480 controls were screened for the p.R1628P variant in LRRK2 and 11% of the PD cohort carried this mutation, which was also related to an earlier disease initiation and a more rapidly progressive course [21].

Finally, a large study from mainland China assessed 1676 unrelated patients with Parkinson’s disease (192 patients from families with autosomal-recessive Parkinson’s disease, 242 patients from families with autosomal-dominant Parkinson’s disease, and 1242 sporadic early-onset PD patients). In total, eight patients harbored pathogenic/likely pathogenic LRRK2 variants, including the p.N1437D, p.R1441C and p.R1441H variants, but not the variant common in Europe, p.G2019S [22]. A second Chinese cohort showed similar outcomes regarding LRRK2 variants, with only 2 out of 832 cases of PD carrying a pathogenic LRRK2 mutation, while p.G2019S was again not detected [23].

LRRK2 mutations were relatively rare in Indians. In the study of Vishwanathan and co-authors, after evaluating 140 Indian PD patients, only one harbored the G2019S LRRK2 mutation, while other studies failed to identify any carriers [24]. Furthermore, in a large number of Indian patients (*n* = 626) with Parkinsonian disorder, another group managed to identify only one patient carrying the p.Gly2019Ser LRRK2, thus implying a limited role of LRRK2 mutations in Indians [25]. Recently, Kishore and co-authors performed targeted sequencing of the LRRK2 locus in 288 PD patients and 298 controls and identified four novel missense variants for LRRK2, which are considered to be specific to the Indian population [26].

LRRK2 mutations appear to be ultra rare or absent in Black African population studies. In two Nigerian population studies (a modest cohort of Sub-Saharan African PD patients (*n* = 126) and controls HC (*n* = 54) and a second cohort of 92 Nigerians with PD and 210 HC), all patients and controls were negative for the p.G2019S mutation and other rare LRRK2 pathogenic mutations described in Caucasians, Asians, and persons of mixed ancestry [27,28]. In a South African study, G2019S LRRK2 was not found in any of the Black PD patients assessed. Moreover, four South African G2019S-positive probands (three Caucasian and one of mixed ancestry) shared a common haplotype due to European ancestry [29].

In Latin American populations, the genetic admixture appears to determine the genetic background. In the more extensive study up to now, a large number of participants (PD patients *n* = 1734 and HC *n* = 1097) in the Latin American Research Consortium on the Genetics of Parkinson’s disease (LARGE-PD) were screened for common pathogenic variants. There was a considerable variation in the presence of LRRK2 p.G2019S in different Latin American countries (from 0.2% in Peru to 4.2% in Uruguay). Such differences could be partially explained by the extent of European ancestry observed in each country. Furthermore, according to this study, p.R1441G is rare in Latin America, even in patients of Spanish ancestry, as one case found, had a different haplotype possibly owing to mixed ancestry [30]. No known pathogenic LRRK2 mutations could be identified in a study assessing the Costa Rican PD population (Clinical and Genetic Analysis of Costa Rican PD population) [31].

#### 3.1.2. SNCA

The identification of the p.A53T mutation in the α-synuclein (SNCA) gene was a milestone of PD genetics research [4]. Since 1997, additional missense SNCA mutations have been reported [1]. Moreover, multiplication mutations like SNCA duplication or triplication have been described. These mutations result in a gain of function that enhances the aggregation of the insoluble form of the protein and exerts a deleterious action likely at the level of the synapse and lysosomal function [32]. The p.A53T mutation was found in members of Italian or Greek families, possibly as a consequence of a common founder effect [4,33] and subsequently in a limited number of patients from other countries. The p.E46K mutation has been described in members of a family of Spanish Basque background [34]. Other variants, including p.A30P and p.G51D, have been reported in patients from different countries like Germany [35] and can have a variable atypical presentation. The phenotype of SNCA point mutations is largely heterogeneous. Non-motor features are common, including olfactory and autonomic dysfunction. RBD, depression or cognitive decline may also be often present [1,36]. SNCA triplications cases exhibit more severe symptoms, including concomitant cognitive decline and an early disease onset [37]. SNCA locus duplications have a variable clinical phenotype [38]. Non-motor symptoms, like dysautonomia, depression, or psychosis, were reported in approximately half of the reported cases.

Pathogenic SNCA variants were either rare or absent in most populations of non-European ancestry (Table 1). A study on the early-onset PD of the Kazakhstan population failed to identify any pathogenic SNCA mutation [16]. The p.A53T SNCA mutation has been identified in a single case of a Han Chinese PD patient [39]. In the large 2020 Chinese screening study, four probands carried SNCA pathogenic/likely pathogenic variants, including three patients with SNCA duplications and one patient with an unreported missense variant (p.P117S) [22]. A common SNCA pathogenic variant, p.A53V, identified in a Chinese cohort, occurs exclusively in Asians [40]. Moreover, the p.A53T SNCA mutation was identified in a Korean PD case with autosomal-dominant inheritance and subsequently in another case from the same family in a population study in Korea [41]. Interestingly, this occurred on a different haplotype than the one originally reported and thus likely represents an independent mutational event. 

In a Japanese study, symptomatic patients with SNCA duplication (2 out of a screened population of 113 autosomal-dominant PD patients) showed abnormal olfactory function and RBD in polysomnography [38]. Another duplication carrier from Japan presented a peculiar phenotype with atypical shaking movements of the head [42]. A Japanese family harboring SNCA triplication has also been described [43].

To our knowledge, no SNCA mutation could be identified in three screening studies in the Indian population [24,44,45].

As far as Sub-Saharan Africa is concerned, pathogenic mutations were not detected in SNCA in a study screening 39 Zambian iPD patients [46], and the same was true in another cohort of black South African patients with PD [47]. Furthermore, a cohort of 109 Nigerian patients with early-onset PD were screened for known pathogenic variants and SNCA point or dosage mutations could not be identified [48].

Regarding Latin American populations, no SNCA pathogenic variants were reported in a study assessing the population of Costa Rica [31]. Moreover, in a cohort of 141 cases from families with apparently dominant PD inheritance in Brazil, no SNCA mutations could be identified [49].

#### 3.1.3. VPS35

Variants in the gene regulating the vacuolar protein sorting 35 ortholog (VPS35) protein are considered to be another cause of monogenic PD [1]. Affected carriers have typical PD symptoms, much like LRRK2 carriers, apart from an early PD initiation in some cases. In terms of pathophysiology, mutations in VPS35 impair vesicular recycling [50]. When first described, the mutation p.D620N was found in Austrian and Swiss families [51]. Clinically, PD patients harboring this mutation exhibited symptoms typical of idiopathic PD. No cognitive problems were noted except for a few patients with mild cognitive impairment. Moreover, the olfaction was decreased in about half of the subjects, and rarely, neuropsychiatric features have been described [51].

Apart from some large pedigrees identified mainly in Germany and central Europe, this mutation seems to be scarce despite the fact that a few sporadic cases have been reported (Table 1) [52].

A Japanese group reported seven missense variants of VPS35, p.D620N being the most important, in a screening effort of 300 Japanese patients with autosomal-dominant PD and 433 patients with idiopathic PD and yielded three p.D620N mutation carriers with autosomal-dominant PD (1.0%) and 1 patient with sporadic PD (0.23%) harboring the same mutation. According to this report, the prevalence of VPS35 is higher among Japanese compared to other Asian populations [53].

In a large Chinese study no VPS35 gene c.1858G > A (p.Asp620Asn) mutation could be detected in a sample of 1011 sporadic PD patients and 1016 controls. The authors suggested that the VPS35 variants are not associated with PD in the mainland Chinese population [54]. Accordingly, in a recent large study from mainland China, assessing 1676 unrelated patients with Parkinson’s disease, only one VPS35 mutation carrier was found [22].

In an Indian PD population study assessing 250 patients, a novel VPS35 variant was identified [55]. Sudhaman and co-authors could not identify any VPS35 pathogenic variant in another Indian cohort [56].

VPS mutations are absent in Sub-Saharan Africans, as exemplified by the screening of South African PD patients and HC of diverse ethnicities (*n* = 418 and *n* = 528, respectively) for the D620N mutation in the VPS35 gene (no carriers were found) [57]. Similarly, in a cohort of 141 unrelated PD cases in Brazil, no VPS35 mutations could be identified, highlighting the rarity of such mutations in Latin America [49].

### 3.2. Autosomal-Recessive Genes

#### 3.2.1. PRKN (Parkin)

Pathogenic variants in the PRKN gene, which encodes for Parkin, represent a major genetic cause of early-onset PD [1]. Such variants were initially reported in patients from Japan with an autosomal recessive inheritance pattern [58]. Genetic changes are either missense or nonsense mutations, but they can often be Copy Number Variants (CNVs). It is apparent that these mutations cause a loss of function. Parkin is an E3 ligase whose role in the Ubiquitin–Proteasome System is crucial and results in the degradation of certain substrates. It is highly possible that PRKN mutations are linked to PD via mitochondrial dysfunction (in mitophagy which involves the inactivation of damaged mitochondria by means of autophagy) [59]. Pathology assessment in Parkin-related PD showed neuronal loss in substantia nigra and locus coeruleus, but no Lewy body pathology was evidenced. The phenotypic spectrum involves slow motor progression, frequent concomitant dystonia (mainly prominent in the lower limbs) with gait abnormalities and early-onset dyskinesias and motor fluctuations [60]. Cognitive function is not affected even in later disease stages [60], but psychiatric manifestations, including depression, anxiety, obsessive–compulsive symptoms, panic attacks or, occasionally, psychosis, may occur. We should highlight the fact that the pathogenicity of monoallelic PRKN mutations as a risk factor for PD remains elusive. Most recent studies, however, do not support a pathogenic role of heterozygous PRKN variants [61].

PRKN pathogenic mutations appear to have a widespread distribution across the globe (Sub-Saharan Africa, East and Central Asia, India and Latin America) (Table 2). PRKN pathogenic mutations were present in Central Asian populations in Kazakhstan (1 patient out of 50 young-onset PD carried a homozygous pathogenic PRKN p.(Arg84Trp) variant) [16].

In East Asia, PRKN was particularly common in young-onset PD in mainland Chinese studies, in Taiwan, Japan and Korea. In the large Chinese 2020 cohort, PRKN was the most prevalent pathogenic variant, as 50 out of 192 patients with an autosomal-recessive inheritance pattern (26.04%) were carriers (27 PD patients harbored homozygous and 23 compound heterozygous variants). The prevailing form of mutations appeared to be exon deletions, mostly in exon 3 and exon 4. Additionally, 21 probands carried PRKN point mutations (7 p.G284R and 3 p.M1T). PRKN mutations were also the most common genetic finding in the sporadic early-onset cohort, accounting for 33 out of 1242 patients (2.66%) [22]. Moreover, in a study performed in Taiwan, 4 out of 41 young-onset PD patients carried PRKN mutations (including deletions in exons 2 and 3 and point mutations like G284R in exon 7) [62]. In a second Taiwanese study, PRKN was the most common pathogenic variant in 324 patients with early-onset PD (EOPD). Four out of the fourteen PRKN mutation carriers (4.3% of EOPD) harbored compound heterozygous mutations, and ten carried heterozygous mutations. The most frequent type of mutations were exon deletions (ins exon 2, 3 and 5, whereas six probands carried point mutations (3 p.C441R, 2 p. R396G and 1 p.Y267H variant) [63].

Regarding other Asian populations, a study assessing the genetic landscape of PD in Malaysia showed comparable outcomes, as 11 out of 161 young onset PD patients (6.8%) harbored PRKN mutations. PRKN exon 7 deletion appeared to be common among Malay patients [64]. In a Vietnamese early-onset PD population (N = 83), among 24 pathogenic/likely pathogenic mutations found in this study group, the second most common altered alleles were detected in PRKN (second to LRRK2), corresponding to 29%. [20]. A study in Japan enrolled 221 patients (26 with a positive family history of PD), and the authors concluded that, in the Japanese PD population, PRKN and LRRK2 were the most prominent causative genes [19]. Finally, a recent study in the Japanese PD population recruited 2322 patients (1204 with positive family history and 1118 with sporadic PD), and the research group managed to identify 242 patients harboring PRKN variants (either heterozygote or homozygote/compound heterozygote) [65].

In the Indian subcontinent, PRKN mutations are often encountered in the genetic analysis of PD cohorts. An assessment of 97 patients, including 52 sporadic early-onset PD and 45 familial PD, revealed a total of 25 (25.77%) patients carrying PRKN variants (5 homozygous/compound heterozygous and 20 heterozygous mutations only) [66]. In another Indian PD population study assessing 250 patients, 21 carried known pathogenic variants in recessive genes, and a novel PRKN variant in a homozygous state was identified [55]. Chaudhary and co-authors reported a prevalence of PRKN gene mutations in 8.5% of the PD population in India. They failed to find any homozygous deletions, but 9.2% of patients had heterozygous exon rearrangements [67]. Similarly, Biswas and co-authors identified PRKN mutations in 7.24% of Indian PD patients cases (25% of cases with autosomal recessive inheritance carried a PRKN mutation) [68]. In the Northwestern Indian PD population, screening for PRKN mutations revealed the occurrence of homozygous deletions in 28/69 patients assessed (40.5%) [69].

PRKN mutations are occasionally found in Black African PD patients. Four PD cases harboring PRKN mutations have been described: two from South Africa carrying a heterozygous PRKN duplication in exon 2 and an exon 9 deletion; a heterozygous PRKN deletion in exon 4 and a point mutation, respectively [70,71]. Moreover, a patient from Zambia carried a heterozygous PRKN exon 2 and a PRKN exon 4 deletion [46], and a patient from Nigeria harbored a homozygous PRKN exon 4 deletion [72]. A study group in Nigeria assessed 109 patients with PD from the four prevalent tribes in the country. Fifteen EOPD patients were identified, and 9/15 (40.9%) harbored heterozygous PRKN variants but none carried homozygous or compound heterozygous variants [48].

In Mexican PD patients, PRKN accounted for the majority in early-onset PD cases. Monroy-Jaramillo and co-authors screened 127 patients and 120 Mexican Mestizo HC for the three commonest recessive PD genes (PRKN, PINK1 and DJ-1). Among them, 10 carried PRKN mutations and interestingly two PARK2-PINK1 and one PARK2-LRRK2 digenic cases were detected. Exon (9 and 12) dosage deletions were the most common mutations found in PRKN [73]. In another study of the Mestizo population in Mexico, PRKN mutations were detected in 34 out of 63 early-onset PD patients (54.0%). In this study, 32 patients carried exon rearrangements, (mainly in exons 9 and 12), including 17.5%, which carried simple heterozygous and 25.4% compound heterozygous PRKN variants [74]. Finally, a Latin American study examined 426 PD patients from Ecuador and 80 from Colombia for PD-related genes. No PRKN carrier could be identified among Ecuadorians, but in the Colombian population, the research team identified eight variants in PRKN (including a PRKN exon 5 and 6 duplication, a PRKN exon 5 and 6 homozygous duplication, and a PRKN exon 7 homozygous deletion, each in different patients) [75].

#### 3.2.2. PINK1

After Parkin, PTEN-induced putative kinase 1 (PINK1) mutations are considered to be an important causative gene of young-onset PD worldwide and account for 1–5% of cases. As in the case of PRKN, there is variation as related to ethnicity [1]. Point mutations or dosage mutations (small deletions/insertions) have been described in a compound heterozygote or homozygous state [76]. There is evidence that the mechanism of action involves mitochondria. Its exact role is still obscure, but it possibly has a crucial role in mitophagy [77].

Regarding a patient’s phenotype, Ishihara-Paul (2008) evaluated sporadic and familial Tunisian PD cases [78]. PINK1 carriers often have a rather early age of PD onset. From a clinical perspective, these patients have a rather benign clinical course but present more often with difficulties in gait and balance. Cognition is less affected compared to other genetic PD causes. Again, as the case is with PRKN variants, the importance of monoallelic PINK1 mutations as a risk factor for PD remains elusive [79].

PTEN-induced kinase 1 (PINK1) has been reported to be an important recessive PD gene after PRKN in sporadic and familial PD in East Asia (China, Korea) and India (Table 2). A group of Malaysian PD patients from multiple ethnic backgrounds (185/499 were Malays) revealed five cases of homozygous p. Leu347Pro variants, resulting in a frequency of 6.9% among Malays with EOPD [80]. PINK1 is a rare cause of early-onset PD in Japan, as a recent study assessing 1700 patients (842 with positive family history and 858 sporadic PD patients from Japan) reported thirty patients carrying heterozygous, three homozygous, and three digenic variants of PINK1-PRKN [79]. In a large 2020 Chinese cohort, PINK1 was rarer than PRKN since five patients harboring homozygous PINK1 pathogenic/possibly pathogenic variants and one patient harboring a compound heterozygous PINK1 mutation were detected in cases with autosomal-recessive inheritance, while only one case was found in sporadic PD [22]. In a Korean study, 70 patients with EOPD were screened for PD-related genes; three rare variants in PINK1 were detected, two of which were likely pathogenic [81]. In a study among the Filipino population, five individuals were carriers of the PINK1 c.10140T > C(p.L347P) variant, whereas one had a heterozygous PRKN c.136G>T(p.A465) mutation [82]. Finally, in a multiethnic PD group from Asia, including ethnic Chinese, Malays, and Indians residing in Singapore, three different variants (two homozygous nonsense and one heterozygous missense) were identified in three patients, indicating a 3.7% prevalence of PINK1 variants in this population [80].

In the Indian population, out of 250 PD patients screened, two putative pathogenic mutations (het Arg246Gln and het Arg276Gln) were detected in five patients, though none harbored homozygous/compound heterozygous variants [83].

A study group in Nigeria assessed 109 patients with PD from the four prevalent tribes in the country. Out of 15 patients with EOPD, 10 PINK1 variants were detected; only three rare heterozygous variants were reported (13.6% in this population) [48]. Moreover, a cohort of in 154 PD patients from South Africa (mixed European and Black African ancestry) was screened for PINK1 variants. A number of 16 PINK1variants were found, including one already described homozygous variant (Y258X), two variants in a heterozygous state (P305A and E476K), and 13 additional polymorphisms [70]. In a Zambian study, no PINK1 variants could be detected [46].

Monroy-Jaramillo and co-authors screened 127 patients and 120 Mexican Mestizo HC for the three commonest recessive PD causative genes (PRKN, PINK1 and DJ-1). Among them, six carried PINK1 mutations, and two PARK2-PINK1 digenic cases were observed. They mostly reported dosage mutations [73]. Finally, in the Latin American study mentioned above, researchers assessed 426 PD patients from Ecuador and 80 from Colombia for PD-related genes. No PINK1 carrier could be identified among Ecuadorians, but in the Colombian population, the research team identified three pathological PINK1 variants (p.L63L, p.A340T, p.N521T) not previously reported in Latin America [75].

Finally, a biallelic p.L347P PINK1 variant has been identified in 12 Polynesians as a cause of EOPD with mixed features of parkinsonism and/or L-Dopa-responsive dystonia [84,85].

#### 3.2.3. DJ-1

DJ-1 represents a scarce recessive type of early-onset PD. DJ-1 protein is important in the anti-oxidant response and possibly shares a biological pathway with PINK-1 and Parkin [1,86]. A homozygous deletion involving DJ-1 was initially reported in the Netherlands, while a homozygous point mutation, p. L166P, was described in a family from Italy [86]. The clinical features resemble Parkin-related PD, with early age of onset, occasional dystonic symptoms, marked L-Dopa-induced motor fluctuations, and anxiety or depression [87].

Parkinson disease protein 7 (DJ-1) has been described rather rarely in East Asia (China, Taiwan, Malaysia and Korea) (Table 2). In a Chinese study, 3 out of 127 (2.4%) unrelated early-onset PD patients carried possibly pathogenic DJ-1 variants [88]. Zhao and co-authors reported only one familial DJ-1 case (possibly a pathogenic variant) with autosomal recessive inheritance in a large Chinese cohort [22]. In a Korean study, 70 patients with EOPD were assessed for PD-related genes, and a pathogenic variant in DJ-1 was detected [81]. Notably, the evaluation of a Taiwanese cohort (68 probands, of which 58 were sporadic and 10 were familial) with early-onset parkinsonism failed to detect any DJ-1 mutation carriers [89].

In the Indian population, the screening of early-onset PD patients for DJ-1 revealed a first-reported homozygous variant (c.313 A > T, p. Ile105Phe) in a patient and an additional polymorphism rs71653619 (c.293 G > A, p.Arg98Gln) in four unrelated patients (prevalence of DJ-1 related EOPD approximately 5%) [90]. Moreover, according to the study of Sadhukhan and co-authors, DJ-1 variants might be present in approximately 3.9% (12 out of 308) of eastern Indian PD patients [91].

No pathogenic DJ-1 mutations have been reported for Black African or Latin American populations [46,71].

### 3.3. GBA1

GBA1 gene mutations represent a common genetic predisposing factor for PD. Glucocerebrosidase (GCase) (the protein product of the GBA1 gene) is a lysosomal enzyme that degrades glucosylceramide into glucose and ceramide. A multitude of GBA1 variants have been identified, with the majority causing severe attenuation of glucocerebrosidase activity [1]. Except for its role in Gaucher’s disease, GBA1 mutation heterozygotes lead to an increased incidence of PD [92]. The proposed causative mechanisms of PD comprise the aggregation of α-synuclein and the dysregulation of the homeostasis of lysosomes. GBA1 mutations result in an earlier PD initiation by about 1.7–6.0 years [92,93]. Brockmann et al. (2015) reported that PD-GBA1 had more intense motor deterioration in spite of their early disease age vs. non carriers [94]. Moreover, longitudinal intra-group evaluation revealed a more rapid course of decline in cognition in GBA1 PD carriers. Psychiatric symptoms, including psychosis, may also be more pronounced in GBA1 carriers. Finally, other non-motor symptoms (like RBD, hyposmia and autonomic dysregulation) seem to be at least as prominent as in idiopathic PD [95]. GBA1-associated PD has a worldwide distribution but carriers are especially frequent amongst Ashkenazi Jews, accounting for 15% of PD cases, mainly carrying the N370S variant [92].

Glucocerebrosidase gene variants (GBA1) were found in over 5% of Asian cases [Mainland China, Taiwan, Korea and Thailand (mostly the p.L444P, RecNcil and R120W while the N370S was rare)] (Table 1). GBA1-related PD was rather prevalent in the Chinese population. Yu and co-authors evaluated a Chinese cohort (184 PD and 130 HC) by means of complete sequencing of the GBA1 gene. The prevalence of GBA1 variants in this group was 8.7%. Identified variants included three novel (5-bp deletion (c.334_338delCAGAA), L264I and L314V) and nine already reported GBA1 variants (R163Q, F213I, E326K, S364S, F347L, V375L, L444P, RecNciI and Q497R) [96]. In another Chinese study, out of 737 PD patients assessed, GBA1 variants were found in 79 patients (10.72%). The most notable GBA1 mutations identified were R163Q, L444P, and R120W. Overall, 18.50% of PD carried a GBA1 mutation [97]. A study in a Taiwanese population showed that the L444P mutation in the GBA1 gene may result in early PD initiation in the Han/Chinese population and that GBA mutations may be considered as the second most frequent genetic cause of sporadic PD development in this population. In this study, 967 PD and 780 HC were screened, and researchers reported that 3.72% of PD harbored a pathogenic mutation in GBA1 (27 L444P, 7 RecNciI, and 2 D409H) [98].

In a large Japanese study, the screening of 534 PD participants and 544 HC for GBA1 variants revealed 50 PD carriers of a GBA1 variant (9.4%), with R120W and L444P/RecNciI being highly prevalent [99]. The odds ratio for GBA1 mutations in PD compared to controls is close to 30 in Japan [90].

GBA1 mutations are also present in patients of Malaysian origin (with the L444P mutation being the commonest). A large study of a multiethnic PD population in Malaysia evaluated the spectrum of GBA1 variants. In this study, 496 patients were screened (*n* = 496) belonging to Chinese, Malay, and Indian ethnical backgrounds. The researchers managed to identify 14 heterozygous GBA alleles in 25 patients (5.0%). The most common variant was p.L483P [p.L444P] (including RecNciI, (2.2%)), found in all groups along with newly described variants. However, the usual mutations found in Europe, p.E365K, p.T408M, and p.N409S (N370S), were not reported [100]. GBA1 mutations also represent an important risk of PD in the Thai population. A group of 108 EOPD patients and 100 PD with disease initiation over 50 years were evaluated for GBA1 mutations. Heterozygous GBA1 mutations were detected in 24 patients (5%) The seven identified GBA1 point mutations included p.L444P, p.N386K, p.P428S, IVS2+1G > A, IVS9+3G > C, IVS10-9_10GT > AG and c.1309delG, of which five variants had not been previously described [101].

GBA1 variants have also been found in a considerable number of cases in India, mainly the L444P, and other variants rare in Europeans. Kukkle and co-authors analyzed whole-genome sequencing data from a cohort of 90 young-onset Parkinson’s disease and found GBA1 heterozygous variants in 13 individuals (14.4%) [including E365K, L483P (L444P), R159Q, D448H, P454L, R502P and R502H variants] [102]. Another study assessed an eastern Indian population, with the patient cohort consisting of 198 typical PD cases and 136 PD cases with concomitant dementia. They reported only the p.L444P variant in nine PD cases [103].

GBA1 mutations have been rarely identified in Black African populations (with different variants as opposed to common risk GBA1 variants in Europeans like N370S). In a study on thirty South African PD patients of black ancestry, all 11 exons of GBA1 were evaluated, and the research group found two carriers of pathogenic mutations (p.R120W in and p.R131L) [104]. Furthermore, in a Nigerian population study, the screening of GBA1 mutations revealed six putative pathogenic mutations only in patients (p.W184R, p.L383PfsX3, and 3 p.L444P) (6.5% of PD cohort) [105]. At this point, we should highlight the fact that a very recent extensive genome-wide association study (GWAS) in people of African and African mixed ancestry with and without PD managed to identify a novel risk factor in GBA1 at the rs3115534 g locus in people of African ancestry. This non-coding variant (found in 39% of Sub-Saharan PD patients) seemed to be associated with reduced Glucocerebrosidase activity, which was absent in European populations and could, consequently, constitute a novel disease-causing mechanism for PD in African populations. This discovery, in particular, highlights the value of performing genetic studies in PD cohorts that are diverse ethnically, beyond Caucasian populations [106].

Latin American populations of indigenous/mixed origin have been reported to harbor different GBA1 variants than those encountered in the population of European ancestry. In a study of Colombian and Peruvian populations, 602 PD and 319 HC were screened by sequencing the entire GBA1 coding area in the context of the Latin American Research Consortium on the Genetics of Parkinson’s disease (LARGE-PD). The authors identified a higher percentage of GBA1 mutation carriers in patients (5.5%) compared to healthy controls and, notably, the presence of variants in patients from Colombia (9.9%) was more than double that in patients from Peru (4.2%), possibly due to an ethnicity-specific GBA1 variant (p.K198E) encountered exclusively in the Colombian cohort [107]. Finally, a Brazilian study group reported a relatively high prevalence of established pathogenic variants in the GBA1 gene among the EOPD cases assessed (6 out of 110, 5.4%) [108].

### 3.4. Other Rare Genes

Mutations in additional rare genes have been described in Chinese and East Asian studies (CHCHD2, ATP13A2, PLA2G6, EIF4G1 and FBX07). Phospholipase-associated Neurodegeneration (PLAN) with PLA2G6 mutation involves a spectrum of three distinct clinical manifestations, including PLA2G6-associated dystonia-parkinsonism. In a Chinese study, variants in PLA2G6 were not particularly rare and accounted for 1.89% of early-onset PD [23]. An Indian group described juvenile PLA2G6-Parkinsonism due to a p.R741Q mutation [109]. A Chinese population study showed that the Pro2Leu variant in CHCHD2 may be related to the development of PD among Asians [110].

## 4. Discussion

The accumulated data reviewed in our current report support the idea that the landscape of monogenic PD forms is heterogeneous among different populations. This applies especially to relatively common pathogenic variants in European ancestry populations (including the p.G2019S in LRRK2 and the p.N370S in the GBA1 gene). SNCA mutations were almost absent and observed rarely in Central and East Asian cases. East Asian patients often harbored different LRRK2 variants than European ancestry populations, with the p.G2019S mutation being especially rare. Parkin (PRKN) variants exhibited global distribution and were commonly encountered in EOPD in East Asians, in Indians and in early-onset PD of Mexican ethnicity. Other recessive genes like PINK1 and DJ-1 were occasionally reported in East and Southeast Asian, Indian and Pacific Islands populations. GBA1 variants were also widely distributed as a predisposing factor for PD and were found in East Asian and in Indian populations, whereas different GBA1 variants have been occasionally reported in Latin Americans. In Black African populations, a recently described novel GBA1 variant with a different proposed mechanism of action might play a crucial role in PD genetics [106].

The geographic variation of PD genetic diversity between populations is regarded as an outcome of a balance between factors causing local genetic variation and gene flow between different populations. The relative isolation of such populations in the previous centuries, as in the case of Sub-Saharan Africa and indigenous Latin America, may explain the marked genetic differences in PD-related genes in these populations.

Our review is the first to assess genetic PD causes from a worldwide perspective despite previously published reviews on specific regions (like Sub-Saharan Africa, Latin America or East Asia). Moreover, a more profound knowledge of the genetic background of PD is increasingly recognized as a cornerstone of personalized medicine. The genetic profile should be considered for individualized treatment options, including the selection of symptomatic therapy, more targeted device-aided treatments and even for inclusion in emerging clinical studies that are applied to specific genetic forms of the disease. Potential limitations of our review may include the relative paucity of PD genetic data for specific populations as is the case for Black Africans and indigenous South Americans.

Despite these insights, the majority of non-European ancestry patients remain underrepresented in clinical studies, even including people of non-European ancestry residing in Europe and North America [5]. The necessity for increasing diversity in PD studies is thus more than evident. Large-scale genetic assessments in Asian, African, or Latin American populations, which are ongoing, will probably inform clinical studies [5]. The Global Parkinson’s Genetics Program (GP2), the Michael J. Fox Foundation (MJFF) Global Genetic PD Cohort, the Latin American Research Consortium on the Genetics of Parkinson’s Disease, the International Parkinson’s Disease Genomics Consortium Africa, the Genetic architecture of PD in India (GAP-India) study, the Luxembourg-German-Indian Alliance on Neurodegenerative diseases and Therapeutics (LUX-GIANT) network and similar initiatives aim to incorporate populations of diverse genetic background in PD genetic research [111,112,113,114,115,116,117,118]. The outcomes of such efforts are already available and will probably inform future clinical studies. The ultimate target is the identification of either new pathogenic mutations in already described genes or novel causative PD genes, thus contributing to the concept of personalized medicine and advancing the understanding of PD pathogenesis.

## Figures and Tables

**Table 1:** Variation in monogenic PD genetic background (autosomal-dominant) according to geographical/racial distribution.

Gene	East Asia (incl. China)	Central Asia	India	Sub-Saharan Africa	Latin America	Pacific Islands
α-synuclein (SNCA)	Very scarce; the p.A53T mutation reported on a different genetic background in Korea	Very scarce	N/A	N/A	N/A	N/A
Leucine Rich Repeat Kinase 2 (LRRK2)	Mainly region-specific variants like G2385R, R1628P. R1441C/G/H in China and p.I2020T in Japan. Additional variants in Thailand and Vietnam	G2019S in 5.7% of PD	Rare	Almost absent, only in mixed ancestry cases (South Africa)	R1441G/G2019S were present in mixed ancestry cases	
Vacuolar protein sorting 35 ortholog protein (VPS35)	The prevalence of VPS35 is higher among Japanese as compared to other Asians	N/A	Very Rare	N/A	N/A	N/A
Glucocerebrosidase gene variants (GBA1)	In total, 5% or more of Asian cases [Mainland China, Taiwan, Korea and Thailand (mainly the p.L444P, RecNcil and R120W)]. In Malaysia, mainly L444P. N370S is rare.		GBA1 variants found in 5–14% of cases, mainly the L444P and other variants (some rare in Europeans)	Novel genetic variant in GBA1 as a risk factor in people of African ancestry (present in 39% of cases).	Occasionally reported in indigenous Latin Americans, those of mixed ancestry had variants common in Europeans	

**Table 2:** Variation in monogenic PD genetic background (autosomal-recessive) according to geographical/racial distribution.

Gene	East Asia (Incl. China)	Central Asia	India	Sub-Saharan Africa	Latin America	Pacific Islands
Parkin (PRKN)	Common in young-onset PD in mainland Chinese studies in Taiwan, Japan, Malaysia and Korea	Found in Kazakhstan PD patients	In India, PRKN mutations ranged from 7,2% up to 25% in EOPD	Scarce, a few reported cases in Black African PD	In a Mestizo Mexican population PRKN carriers are the majority in early-onset PD	
PTEN-induced kinase 1 (PINK1)	In sporadic and familial of East Asia (China, Korea, Malaysia, and Philippines)		Reported in some cases of early-onset PD	Rare		Biallelic p.L347P PINK1 Variant in Polynesians
Parkinson disease protein 7 (DJ-1)	Rather rare in East Asia (Malaysia, Mainland China, Taiwan, and Korea)		Rare cases	N/A		

## Data Availability

Not applicable.
